# Metabolite Parameters as an Appropriate Alternative Approach for Assessment of Bioequivalence of Two Verapamil Formulations

**Published:** 2014

**Authors:** Azadeh Haeri, Bahareh Javadian, Roonak Saadati, Simin Dadashzadeh

**Affiliations:** a *Department of Pharmaceutics, School of Pharmacy, Shahid Beheshti University of Medical Sciences, Tehran, Iran. *; b *Pharmaceutical Sciences Research Center, Shahid Beheshti University of Medical Sciences, Tehran, Iran.*

**Keywords:** Bioequivalence study, Pharmacokinetics, Verapamil, Norverapamil, High variability

## Abstract

A bioequivalence study of two verapamil formulations (generic verapamil tablets and Isoptin^®^ tablets) was performed by comparing pharmacokinetic parameters of the parent drug and its major metabolite, norverapamil following a single dose administration of 80 mg verapamil hydrochloride in 22 healthy volunteers according to a randomized, two-period, crossover-design study. Moreover, the feasibility of proving bioequivalence of verapamil oral dosing form by means of norverapamil pharmacokinetic parameters was evaluated. Concentrations of verapamil and norverapamil were quantified in plasma using a validated high-performance liquid chromatography (HPLC) with fluorescence detection. The 90% CIs for the log-transformed ratios of verapamil C_max_ (maximum plasma concentration) and AUC_0–∞_(area under the plasma concentration-versus-time curve from time zero to the infinity) were 73 to 101 and 80 to 103, respectively. Similarly, the corresponding ranges for norverapamil were 80-100 and 84-103, respectively. According to the parent drug data, the 90% confidence intervals around the geometric mean ratio of AUC happened to fit within preset bioequivalence limits of 80–125%, whereas those for C_max_ did not. The 90% confidence intervals for both C_max_ and AUC of norverapamil met preset bioequivalence limits. The AUC and C_max _of metabolite, when compared to parent drug, showed a much lower degree of variability and the 90% confidence intervals of the metabolite were therefore narrower than those of the parent drug. These observations indicate that bioequivalence studies using metabolite, norverapamil, could be a more suitable and preferable approach to assess bioequivalence of verapamil formulations due to its much lower variability and therefore lower number of volunteers that are required to conduct the study.

## Introduction

The bioequivalence of two formulations of the same drug implies their equivalence with respect to the rate and extent of absorption and usually involve administration of test and reference drug formulations to 18-36 normal healthy subjects, but patients with a target disease may also be used. In a bioequivalence study, serial samples of biologic fluid (plasma, serum, or urine) are collected just before and at various times after dose administration. While the area under the plasma concentration–time curve from 0 h to the last measurable concentration (AUC_0–t_) (Area under the plasma concentration-versus-time curve from time zero to the last measurable time) generally serves as the characteristic of the extent of absorption, the peak concentration (C_max_, maximum plasma concentration( and the time of its occurrence (T_max_, time at which the maximum plasma concentration occurs( reflect the rate of absorption (1). 

Verapamil, [(R,S)-2,8-bis-(3,4-dimethoxyphenyl)-6-methyl-2-isopropyl-6- azaoctanitrile] is a well-known nondihydropyridinecalcium channel blocker, and it is currently employed in the treatment of angina pectoris, supraventricular arrhythmia’s, hypertension, certain cardiomyopathies and recently as a multi-drug resistance modulator in tumors which express p-glycoprotein. The drug is available in immediate-release, extended-release, and injectable formulations. Oral absorption of verapamil in man averages over 90%, but only 10–20% out of the dose absorbed from the digestive tract penetrates to the circulatory system as unchanged form due to the high hepatic first pass metabolism. Verapamil exhibits bi- or tri-phasic elimination kinetics and is reported to have a terminal plasma half-life of 2 to 8 hours following a single oral dose or after intravenous administration. After repeated oral doses this increases to 4.5 to 12 hours. Considerable inter- and intra-subject variation has been found in plasma concentrations (2-9).It undergoes extensive and variable metabolism in man. Its primary metabolic pathways include N-dealkylation, N-demethylation, and O-demethylation. CYP3A4 is the major enzyme responsible for N-demethylationand formation of the only active metabolite, norverapamil (10, 11). Asnorverapamil plasma concentrations shows a strong positive correlation with the corresponding verapamil concentrations, also a correlation in norverapamil concentration (up to 400 ng.mL^-1^) with the main effect of the parent drug has been reported (12), pharmacokinetic parameters pertaining to this active metabolite may be appropriate for assessing the bioequivalence of parent drug formulations. Furthermore, in the case of drug that is biotransformed to active metabolite, variability in hepatic clearance is very crucial and of particular concern and the parent drug displays a greater variability than the metabolite. As a result a much notably larger group of subjects would be necessary to study bioequivalence with respect to the drug than to the metabolite (13) clearly with ethical and financial drawbacks. All these considerations can be correct for verapamil that following rather high presystemic metabolism transforms to norverapamil.

The aim of our study was to assess the pharmacokinetics and bioequivalence evaluation of verapamil formulation by comparing pharmacokinetic parameters such as AUC_0-∞_)area under the plasma concentration-versus-time curve from time zero to the infinity(, AUC_0-24_, C_max_, T_max_, T_1/2_)Biological half life (and k based on plasma concentration–time values of verapamil and norverapamil after a single oral administration of 80 mg two different verapamil formulations (Isoptin^®^ manufactured by Knoll Pharmaceuticals, Germany, and a generic formulation produced by Sobhan Pharmaceuticals, Iran). We also evaluated the feasibility of studying bioequivalence of different formulations by means of norverapamil pharmacokinetic parameters rather than verapamil. This was done using a randomized, two-period crossover-design with a 1 week washout period in 22 healthy volunteers.

## Experimental

*Chemicals*

Verapamil and norverapamil reference standards were generous gift from Sobhan Pharmaceuticals (Tehran, Iran). α-Isopropyl-α-[(N-methyl-N-homoveratryl)-β-aminoethyl]-3, 4-dimethoxyphenylacetonitrile hydrochloride as internal standard (IS))Internal standard( was purchased from Knoll AG (Ludwigshafen, Germany). Methanol and acetonitrile (HPLC-grade))High-performance liquid chromatography( were from Merck. Isopropanol, *n*-hexane, sulphuric acid and all other reagents were analytical grade and were purchased from Merck (Darmstadt, Germany).

*Study design and volunteers*

The study protocol was approved by the Ethics Committee of Shahid Beheshti University of Medical sciences (Tehran, Iran) and written informed consent was obtained from all volunteers prior to study enrolment. Twelve healthy women (mean age = 24.08 ± 2.84 years; mean body weights = 56.67 ± 5.23 Kg) and twelve healthy men (mean age = 25.75 ± 2.42 years; mean body weights = 70.50 ± 9.94 Kg) participated in this study. No enrolled subjects had any medical problems according to medical history, physical exams, clinical chemistry, complete blood count and urinalysis. Participants were all non-smokers and had not taken medications (including over-the-counter) neither two weeks prior to nor during the study period. A double-blind, randomized, cross-over design was used for this study. Volunteers were randomly assigned to receive orally 80 mg of verapamil hydrochloride along with 150 mL of water either as a test or reference product. Both treatments were administered under supervision following an overnight fast of at least 12 h, and subjects continued to fast for at least 2 h after dosing. Subjects were given standard breakfast and lunch 2 and 5 h following the drug intake. Each volunteer received both test and reference product with at least 7 days washout between treatments and therefore everyone served as his or her own control. 

The systolic and diastolic pressure and pulse rate were determined before and 1, 2, 3, 5 and 9 h after dosing. Two subjects from test group withdrew due to personal reasons. So only the twenty-two volunteers, who had completed the study, were fully evaluated for bioequivalence assessments.

*Blood sampling*

To determine the plasma concentration of verapamil and its primary metabolite norverapamil, 5 mL of whole blood was drawn from each of the subjects. The time-points at which blood was collected in each case were immediately before (0 h) and 0.25, 0.5, 0.75, 1, 1.5, 2, 2.5, 3, 4, 5, 7, 9, 11 and 24 h after administration of each verapamil formulation. Plasma was separated just after sample collection and was frozen at -20 ºC for subsequent evaluation.

*Analytical assays*

Plasma samples were analyzed for verapamil and its main metabolite norverapamil using a validated high-performance liquid chromatography (HPLC) method with fluorescence detection (λ_excitation_=280 nm, λ_emission_=320 nm) as described previously (14). Plasma samples were defrosted at room temperature. Sample preparation was done by liquid phase extraction with 6 mL of a mixture of n-hexane-isopropanol (97.5:2.5) from alkalinized plasma (1 mL) to which 50 µL of IS solution (500 ng.mL^-1^) had been added. Following shaking extraction tubes for 15 minutes, they were centrifuged at 3000 g for 5 minutes. Subsequently, the organic phase was separated and the sample was re-extracted using 500 µL of 0.02 N sulphuricacid. After mixing and centrifugation, 100 µL of the acidic phase was injected into the HPLC column (Novapak C18, 4 µm, 250×4.6 mm, Waters, MA). The mobile phase consisted of acetonitrile and 0.05 M potassium dihydrogen phosphate buffer (70:30 v/v) adjusted to a pH of 3 with phosphoric acid which was filtered, degassed and pumped at a rate of 1 mL/min through the column at ambient temperature.

*Pharmacokinetic and statistical analysis*

The pharmacokinetics of verapamil and norverapamil in plasma were analyzed using standard noncompartmental methods. First-order elimination rate constant (k) was estimated from the terminal slope of a semi-logarithmic plot of concentration-time data. Half-life of drug elimination during the terminal phase (T_1/2_) was calculated as the ln (2).k^-1^. The areas under the verapamil and norverapamil plasma concentration-time curves from0-24 h (AUC_0-24_) were computed using the linear trapezoidal rule. The AUC_0–∞_ was calculated by dividing the last measured concentration (C_24_) by the k and adding the result to the AUC_0–24_. C_max_ and its associated time (T_max_) were obtained directly from the plasma concentration–time data.

A linear analysis of variance model (ANOVA) was used to analyze the 90.0% confidence intervals (CI) (Confidence interval)for the ratios of the means of the log-transformed pharmacokinetic parameters, C_max_ and AUC_0–∞_of verapamil and norverapamil data. Based on the statistical results, conclusions were drawn as to whether the test product was bioequivalent to the reference product. The test product was then claimed to be bioequivalent to the reference product if the calculated 90% confidence intervals around the ratio of geometric means of the primary study endpoint (AUC and C_max_) were totally within the bioequivalence limits of 80% to 125% (1).

## Results

**Figure 1:**
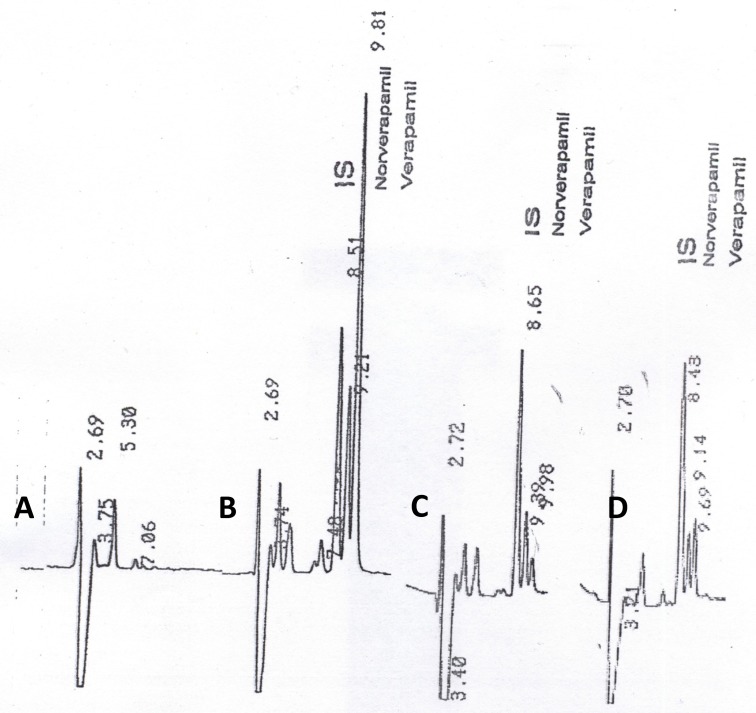
Typical chromatograms of (a) drug-free human plasma, the plasma sample collected from volunteer (b) 0.75 h and (c) 11 h after drug administration and (d) human blank plasma spiked with verapamil (25 ng.mL^-1^).

Under the described analytical conditions, the method was validated over the concentration range of 10 – 200 ng.mL^-1^. Calibration curves were obtained by linear least-squares regression analysis. The assays exhibited linearity between the response (*y*) (peak-height ratio of verapamil or norverapamil over the internal standard) and the corresponding concentration of verapamil or norverapamil (*x*) respectively, over the validated range in plasma. The relationship between *x* and *y* was shown to be linear by the correlation coefficients obtained for the regression lines. The correlation coefficients (*r*) for calibration curves were equal to or better than 0.9996. The coefficients of variation of intra-day and inter-day reproducibility of the assay (at concentrations of 25, 50, 100 and 150 ng.mL^-1^) were less than 4.00%, and 8.5%, respectively. Lower detectable limits for parent drug and its metabolite were 0.30ng.mL^-1^ and 0.35 ng.mL^-1^, respectively. Verapamil, norverapamil and IS eluted separately, without any interference from endogenous substances. Typical chromatograms of human blank plasma, volunteer sample at 0.75 h and 11 h after drug administration and human blank plasma spiked with verapamil (25 ng.mL^-1^)were shown in Figure 1a, b, c and d, respectively.

**Figure 2:**
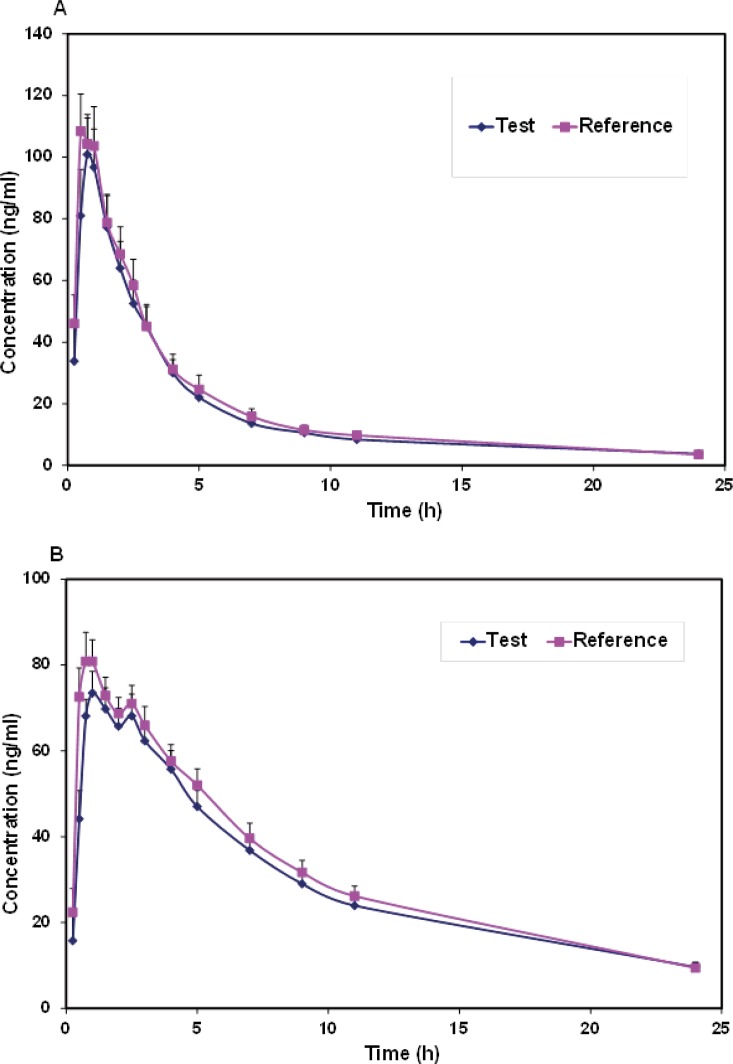
The mean plasma (a) verapamil and (b) norverapamil concentration-time profiles of subjects following administration of test and reference products

Mean plasma concentrations and coefficient of variation (CV))Coefficient of variation( for each formulation at different sample times for verapamil and norverapamil were represented in Table 1. Mean plasma verapamil and norverapamil concentration-time profiles were shown in Figure 2. As shown in comparison to the norverapamil, the plasma concentrations of verapamil displayed a greater variability due to the larger coefficients of variation.

**Table 1:** Mean (CV %) verapamil and norverapamil concentrations (ng.mL^-1^) of the two formulations following a single oral dose of 80 mg (n=22).

**Time (h)**	**Verapamil**		**Norverapamil**	
	**Test**	**Reference**	**Test**	**Reference**
0.25	33.82 (187.5)	45.99 (95.00)	15.79 (179.1)	22.41 (115.6)
0.5	80.98 (87.25)	108.46 (51.58)	44.12 (70.59)	72.55 (43.95)
0.75	100.9 (54.90)	104.29 (43.18)	68.06 (27.39)	80.84 (39.37)
1	96.71 (60.30)	103.60 (57.95)	73.48 (32.12)	80.85 (29.09)
1.5	77.29 (62.03)	78.76 (54.57)	69.74 (33.28)	72.90 (27.30)
2	63.99 (63.86)	68.57 (60.30)	65.74 (29.82)	68.70 (25.49)
2.5	52.48 (68.09)	58.40 (67.78)	68.09 (35.43)	71.01 (28.19)
3	45.15 (73.12)	45.07 (67.20)	62.30 (32.70)	65.93 (31.67)
4	30.03 (66.94)	31.16 (73.92)	55.72 (36.98)	57.60 (31.49)
5	22.06 (69.49)	24.67 (88.00)	47.00 (37.52)	51.98 (34.65)
7	13.61 (64.92)	15.86 (74.46)	36.81 (43.16)	39.57 (43.15)
9	10.61 (66.52)	11.49 (65.01)	29.02 (40.73)	31.68 (41.94)
11	8.41 (63.29)	9.76 (69.37)	23.95 (41.50)	26.17 (41.60)
24	3.78 (75.80)	3.53 (64.48)	9.63 (57.13)	9.43 (59.32)

Both formulations were well tolerated and there were no reports of any significant adverse reactions. Verapamil was rapidly absorbed and extensively biotransformed to norverapamil. As a result, significant plasma concentrations of verapamil and norverapamil were detectable 15 min after dosing and C_max_ values of verapamil and norverapamil occurring 0.54 and 0.96 h following drug administration, respectively (Table 1 and Figure 2). Pharmacokinetic parameters for verapamil and norverapamil from the 22 participants were summarized in Table 2. Following administration of a single dose of 80-mg verapamil, the mean values for verapamil AUC_0-∞_ were 442.2 ng.h.mL^-1^ for the test formulation and 460.6 ng.h.mL^-1^ for the reference formulation. Verapamil and norverapamil C_max_ for test Product is 107.7 and 80.24 ng.mL^-1^, and for reference Product, 122.64 and 89.89 ng.mL^-1^, respectively. The results of comparison between the test and reference AUC_0-∞_, AUC_0-24_, C_max_, T_max_, T_1/2_, and k parameters based on verapamil and norverapamil data revealed no statistically significant difference (P > 0.05).

**Table 2:** Mean (CV %) verapamil and norverapamil pharmacokinetic parameters of the two formulations after a single oral dose of 80 mg (n=22).

**Parameter**	**Verapamil**		**Norverapamil**	
	**Test**	**Reference**	**Test**	**Reference**
AUC_0-24_ (ng.h.mL^-1^)	387.8 (66.44)	412.8 (57.61)	674.6 (37.81)	696.6 (33.81)
AUC_0-∞_ (ng.h.mL^-1^)	442.2 (69.59)	460.6 (56.07)	773.1 (43.02)	823.0 (37.45)
C_max_ (ng.mL^-1^)	107.7 (56.94)	122.64 (44.50)	80.24 (29.50)	89.89 (30.06)
T_max_(h)	0.730 (44.43)	0.540 (35.94)	1.10 (58.73)	0.960 (88.54)
K(h^-1^)	0.093 (76.90)	0.079 (38.89)	0.110 (52.37)	0.077 (38.96)
T_1/2 _(h)	8.610 (59.49)	8.550 (40.94)	8.130 (41.35)	8.42 (36.57)

Analyses of verapamil and norverapamil bioequivalence between two treatments were performed. The analyses focused on C_max_, AUC_0-∞_ and AUC_0-24_values, which were based on approach including classical 90% CI. Ratio of test/reference and the 90% CI for each parameter were calculated for log-transformed data and summarized in Table 3.According to the parent drug data, the 90% confidence intervals around the geometric mean ratio of AUC happened to fit within FDA bioequivalence limits of 80–125%, whereas those for C_max_(0.73 – 1.01) was well outside the accepted limit of FDA. In contrast, for norverapamil the 90% confidence intervals for both C_max_ and AUC were within the bioequivalence limit. The within-subject variabilities (WSVs))Within-subject variability( of C_max_ and AUC of the parent drug were greater than those of the metabolite, and the 90% confidence intervals of the metabolite were therefore narrower than those of the parent drug (Table 3). Clearly, verapamil displayed a greater variability than its metabolite, norverapamil.

**Table 3:** Ratios and 90% CI for AUC_0-24_, AUC_0-∞_ and C_max_ for log transformed data

**Parameter**	**Verapamil**			**Norverapamil**		
	**Log test/reference**	**90 % CI**	**WSV%** a	**Log test/reference**	**90 % CI**	**WSV%** ^a^
AUC_0-24_ (ng.h.ml^-1^)	0.89	0.81 – 1.01	20.44	0.95	0.84 – 1.01	16.85
AUC_0-∞_ (ng.h.ml^-1^)	0.90	0.80 – 1.03	24.49	0.95	0.84 – 1.03	19.94
C_max_ (ng.ml^-1^)	0.85	0.73 – 1.01	34.35	0.90	0.80 – 1.00	21.21

a Within-subject variability expressed as the square root of the residual variance in ANOVA of natural log transformed data.

## Discussion

Bioequivalence defined as: the absence of a significant difference in the rate and extent to which the active ingredient or active moiety in pharmaceutical equivalents or pharmaceutical alternatives became available at the site of drug action when administered at the same molar dose under similar conditions in an appropriately designed study. According to FDA criteria, the two formulations produced by the two manufacturers were bioequivalent when 90% CI limits for the ratio (test/reference) of AUC and C_max_(log-transformed data) fall within 0.8 - 1.25(1).

Verapamil was considered as a drug with highly variable pharmacokinetics (8) mainly because of its high first pass metabolism (15). Under these conditions, even though there were no formulation significant differences, a conclusion of bio-inequivalence between a brand and a generic formulation could draw. As an example, Isoptin SR determined bio-inequivalence against itself in a crossover study in 24 volunteers (8). This definitely resulted in a considerable manufacturer risk (*i.e*. type І error). Midha *et al*. (16) mentioned thatit was improper to increase the producer risk by persisting in the use of a highly variable parameterwhen other parameters of lower variability were available for comparison.

For drugs with considerable first pass effect, the AUC and C_max_ of the parent drug were strongly sensitive to altered clearance. However, T_max_ was not affected. On the other hand, T_max_ and C_max_ of the metabolite were rather sensitive to changes in hepatic clearance but the AUC was not (13). 

Here, our study describes the results of a two-period crossover design in 22 subjects, comparing pharmacokinetic parameters for verapamil and norverapamil following administration of immediate release formulations of test and reference products. The results showed that for the parent drug the 90% CI for the C_max_ did not fall within the standard limit of 80-125%, whereas for norverapamil the 90% confidence intervals for both C_max_ and AUC were within the bioequivalence limit. We demonstrated that the plasma concentrations of the parent drug, verapamil, were much more variable than of the metabolite, norverapamil (Table 1).As indicated in Table 3 the parent drug (verapamil) was highly variable in terms of C_max_ (WSV > 30%), while the metabolite was not highly variable in any measure. The ANOVA-CVs for both C_max_ and AUC of the parent drug were higher than those of the metabolite and therefore the 90% confidence intervals were wider for the parent drug than for the metabolite (Table 3). These results are consistent with a single-dose study on the antipsychotic drug loxapine and two active metabolites (17), the ANOVA-CVs of C_max_ and AUC of the parent drug were greater than those of either metabolite, and the 90% confidence intervals of the metabolite were therefore narrower than those of the parent drug. 

In most cases, the parent drug concentrations were included in bioequivalence assessment (18, 19). A situation for which the use of metabolite data has been advocated is for the bioequivalence of formulations of highly variable drugs. The latter have been defined as drugs with WSV of the maximum plasma concentration and/or area under the plasma concentration vs. time curve of equal to or greater than 30% (20). Very large numbers of subjects are required in the bioequivalence studies to give adequate statistical power when the WSV is high. The use of metabolite data in bioequivalence studies involving highly variable drugs is appealing because metabolites are often less variable than the parent drug such that smaller numbers of subjects are required to achieve statistical power (17, 20). 

Tucker *et al*. (21) performed a simulation study to determine whether the parent compound or the metabolite best predicted bioequivalence as a function of intrinsic clearance. The results showed that until intrinsic clearance exceeded liver blood flow (for drugs with high extraction ratio), the metabolite became a superior predictor of bioequivalence. The probability of concluding bioequivalence was the same for parent drug and metabolite when liver blood flow was higher than intrinsic clearance. When intrinsic clearance approached or exceeded liver blood flow, however, there was roughly a 30% chance of committing a type II error and declaring the test product not to be bioequivalent with the reference product in terms of the C_max_ of the parent drug. For the metabolite, however, depending on the WSV set for renal clearance, there was a 90–100% probability of declaring bioequivalence based on the metabolite in terms of C_max_ (20). 

According to the results obtained in the present study, a large sample size was one of the prerequisites of bioequivalence studies with pharmacokinetic parameters of verapamil which is a drug with high hepatic clearance. In contrast, bioequivalence could be proved with a smaller sample size by analyzing metabolite pharmacokinetics. Hence, it was more economical, eligible and robust to choose metabolite parameters as alternative approach for bioequivalence studies. In a similar study, the bioequivalence of two oxcarbazepine oral formulations was studied through the simultaneous determination of oxcarbazepine and the active metabolite 10,11-dyhydro-10-hydroxy-carbamazepine derivative. The authors demonstrated the bioequivalence of two oxcarbazepine formulations from metabolite pharmacokinetic data of 12 healthy volunteers, whereas it was not possible to prove bioequivalent with parent drug parameters mainly because of higher intra-individual variability of oxcarbazepine than of its active metabolite (22). 

Another reason in our study that suggested norverapamil as an appropriate analyte for the assessment of bioequivalence is that total exposure of norverapamil substantially exceeded that of the parent compound (the verapamil AUC values of 442.2 ng.h.mL^-1^ and 460.6 ng.h.mL^-1^ for the test and reference products, respectively, versus the norverapamil AUC values of 773.1 ng.h.mL^-1^ and 823.0 ng.h.mL^-1^ for the test and reference products, respectively; P < 0.01). It worth to mention that based on the previous reports plasma levels of norverapamil were linearly related to those of the parent compound after various single oral doses of verapamil in normal subjects (23), which suggest linearity in drug conversion to the metabolite.

In conclusion, the role of metabolite in bioequivalence studies for drugs with highly variable drugs like verapamil should not be neglected. Less variable disposition of the metabolite than that of the parent compound was an important advantage for proving bioequivalence while preventing large number of subjects and producer risk.

## References

[1] (2003). FDA Guidance for Industry: Bioavailability and bioequivalence studies for orally administered drugs products-General considerations. US Dept of Health and Human Services, Food and Drug Administration (FDA), Center for Drug Evaluation and Research (CDER).

[2] Eisenberg MJ, Brox A, Bestawros AN (2004). Calcium channel blockers: an update. Am. J. Med.

[3] vanZwieten PA, Pfaffendorf MJ (1993). Similarities and differences between calcium antagonists: pharmacological aspects. J. Hypertens. Suppl.

[4] Echizen H, Eichelbaum M (1986). Clinical pharmacokinetics of verapamil, nifedipine and diltiazem. Clin. Pharm.

[5] Kang D, Verotta D, Krecic-Shepard ME, Modi NB, Gupta SK, Schwartz JB (2003). Population analyses of sustained-release verapamil in patients: effects of sex, race, and smoking. Clin. Pharmacol. Ther.

[6] Thomas H, Coley HM (2003). Overcoming multidrug resistance in cancer: an update on the clinical strategy of inhibiting p-glycoprotein. Cancer Control.

[7] Hamann SR, Blouin RA, McAllister RG Jr (1984). Clinical pharmacokinetics of verapamil. Clin. Pharm.

[8] Tsang YC, Pop R, Gordon P, Hems J, Spino M (1996). High variability in drug pharmacokinetics complicates determination of bioequivalence: experience with verapamil. Pharm. Res.

[9] Kirsten R, Nelson K, Kirsten D, Heintz B (1998). Clinical pharmacokinetics of vasodilators. PartI. Clin. Pharm.

[10] Kroemer HK, Gautier JC, Beaune P, Henderson C, Wolf CR, Eichelbaum M (1993). Identification of P450 enzymes involved in metabolism of verapamil in humans. Naunyn. Schmiedebergs. Arch. Pharmacol.

[11] Busse D, Cosme J, Beaune P, Kroemer HK, Eichelbaum M (1995). Cytochromes of the P450 2C subfamily are the major enzymes involved in the O-demethylation of verapamil in humans. Naunyn. Schmiedebergs. Arch. Pharmacol.

[12] Woodcock BG, Hopf R, Kaltenbach M (1980). Verapamil and norverapamil plasma concentrations during long-term therapy in patients with hypertrophic obstructive cardiomyopathy. J. Cardiovasc. Pharmacol.

[13] Rosenbaum SE, Lam J (1997). Bioequivalence parameters of parent drug and its first-pass metabolite: comparative sensitivity to sources of pharmacokinetic variability. Drug. Dev. Indust. Pharm.

[14] Dadashzadeh S, Javadian B, Sadeghian S (2006). The effect of gender on the pharmacokinetics of verapamil and norverapamil in human. Biopharm. Drug Dispos.

[15] Eichelbaum M, Ende M, Remberg G, Schomerus M, Dengler HJ (1979). The metabolism of DL-[14C]verapamil in man. Drug Metab. Dispos.

[16] Midha KK, Hubbard JW, McKay G, Rawson MJ, Hsia D (1999). The role of metabolites in a bioequivalence study II: amoxapine, 7-hydroxyamoxapine, and 8-hydroxyamoxapine. Int. J. Clin. Pharmacol. Ther.

[17] Midha KK, Hubbard JW, McKay G, Hawes EM, Hsia D (1993). The role of metabolites in a bioequivalence study 1: loxapine, 7-hydroxyloxapine and 8-hydroxyloxapine. Int. J. Clin. Pharmacol. Ther. Toxicol.

[18] Foroutan SM, Zarghi A, Shafaati A, Madadian B, Abolfathi F (2013). Rapid high performance liquid chromatographic method for determination of clarithromycin in human plasma using amperometric detection: application in pharmacokinetic and bioequivalence studies. Iran. J. Pharm. Res.

[19] Mousavi SHH, Kobarfard F; Husain SW, Tehrani MS, Azar PA, Ahmadkhaniha R, Mehdizadeh A (2012). A rapid, simple, liquid chromatographic-electrospray ionization, ion trap mass spectrometry method for the determination of finasteride in human plasma and its application to pharmacokinetic study. Iran. J. Pharm. Res.

[20] Midha KK, Rawson MJ, Hubbard JW (2004). The role of metabolites in bioequivalence. Pharm. Res.

[21] Tucker G, Rostami A, Jackson P, Midha KK, Blume HH (1993). Metabolite Measurement in Bioequivalence Studies: Theoretical Considerations. Bio-International: Bioavailability, Bioequivalence and Pharmacokinetics.

[22] Di Girolamo G, Opezzo JA, Schere D, Gonzalez CD, Moncalvo JJ (2007). Parent drug and/or metabolite? Which of them is most appropriate to establish bioequivalence of two oral oxcarbazepine formulations in healthy volunteers? Expert. Opin. Pharmacother.

[23] McAllister Jr RG, Kirsten EB (1982). The pharmacology of verapamil IV. Kinetic and dynamic effects after single intravenous and oral doses. Clin. Pharmacol. Ther.

